# A predictive equation for operative time estimation in cochlear implant surgery

**DOI:** 10.3389/fsurg.2026.1857136

**Published:** 2026-07-13

**Authors:** Mariam Aljehani, Faris Abdulaziz Albassam, Fida Al-Muhawas, Abdulrahman Hagr

**Affiliations:** 1 King Abdullah Ear Specialist Center, King Saud University Medical City, Riyadh, Saudi Arabia; 2 Department of Otolaryngology–Head and Neck Surgery, King Saud University College of Medicine, Riyadh, Saudi Arabia; 3 College of Medicine, King Saud University College of Medicine, Riyadh, Saudi Arabia

**Keywords:** cochlear implants, intraoperative period, operative time, predictive model, surgical factors

## Abstract

**Objective:**

The study aimed to develop a predictive model for preoperative estimation of CI operative duration by identifying independent clinical and procedural factors that significantly influence surgical time.

**Design:**

Single-centre retrospective observational study.

**Settings:**

King Abdullah Ear Specialist Center (KAESC).

**Participants:**

A total of 216 cochlear implant patients were enrolled between 2019 and 2025.

**Main outcome measures:**

The primary outcome was surgical time (minutes), defined as the duration from skin incision to wound closure. Secondary measures included demographic, clinical, and intraoperative factors evaluated as predictors of surgical duration. Statistical analysis was performed using SPSS version 26 and R version 4.2.2. Univariate and multivariate generalized linear models were used to identify independent predictors of surgical time and develop a final predictive model.

**Results:**

Surgical time was longer in females (180 vs. 150 min, *p* = 0.015), bilateral (190 min, *p* < 0.00001) and primary/revision cases (240 min, *p* = 0.003), and surgeries involving fellows or residents (*p* < 0.00001), whereas the round window approach (*p* = 0.036) and sealing the insertion route (*p* = 0.025) were associated with shorter times. In multivariate analysis, male gender was associated with a reduction in time by 16.6 min with CI 95% (−0.36, −32.77) (*p* = 0.0464), unilateral implantation by 62 min (*p* < 0.00001), and the round window technique by 28.5 min (*p* = 0.0445), while older age was associated with an increase in time by 0.13 min (*p* = 0.0034). A Consultant/Fellow/Resident team added 43 min (*p* = 0.0090).

**Conclusion:**

The developed predictive model is a useful instrument for improving operating room management and customizing care plans in various CI practice environments.

## Highlights

- The round window surgical approach and sealing of the insertion route were linked to shorter surgical durations.
- Operating times are largely affected by gender, age, unilateral implantation, the surgeon combinations, skin incision, and RW technique.
- A predictive equation model was developed, with male gender, unilateral implantation, and round window technique independently associated with shorter surgical times.
- The model offers a simple, internally validated, and consistent alternative to complex machine-learning approaches, outperforming many retrospective studies in clarity and clinical usability.
- A bilingual (Arabic/English) web-based calculator was developed to estimate surgical time using patient and procedural variables, supporting real-time clinical decision-making.

## 1 Introduction

Cochlear implants (CIs) have emerged as the gold standard of care for people with severe to profound sensorineural hearing loss because they significantly improve speech perception and quality of life (1). Due to its widespread use worldwide, the number of CI procedures performed annually is increasing, placing greater strain on surgical teams and operating room resources. Cochlear implantation services in Saudi Arabia have grown significantly over the last two decades, with over 1,000 surgeries performed annually across public, private, and volunteer initiatives, highlighting the need to optimize surgical practices and predict operative times to meet growing demand (2).

However, operational variability in surgical time remains a significant challenge despite advancements in surgical techniques and device designs. A key element of effective healthcare delivery is the precise estimation of surgical duration. Extended surgical durations correlate with heightened anesthetic exposure, perioperative problems, and elevated institutional costs (3, 4). The scheduling of surgeries, the distribution of resources, and patient counseling are thus directly impacted by the discovery of trustworthy preoperative predictors of surgical duration, which is defined as the time from the skin incision to final closure (5). Several factors influence the success of CI surgery, including patient-related factors, surgical techniques, and device characteristics (6, 7). Few studies develop predictive models for the cochlear implantation procedure, lacking clinical granularity due to minor variations in anatomical or technical variables (8). Modern data-driven context-specific research that accurately models CI operative time within contemporary clinical realities is therefore desperately needed.

This study fills the knowledge gap by methodically examining a group of cochlear implantation cases at a tertiary care facility to find important intraoperative clinical and demographic predictors of surgical time. This research aims to offer a useful instrument that can improve surgical planning, maximize operating room productivity, and ultimately lead to better cochlear implantation healthcare delivery.

## 2 Materials and methods

### 2.1 Study design and setting

A retrospective observational study was conducted from 2019 to 2025 at King Abdallah Ear Specialist Center (KAESC), a significant tertiary academic institution that serves as a regional referral hub for cochlear implantation. This manuscript follows the STROBE guidelines for observational studies.

### 2.2 Inclusion and exclusion criteria

The study included CI patients, both adult and pediatric populations. Patients who had either unilateral or bilateral CI and who had fully documented the start and finish times of the surgery (from skin incision to closure) were included. Patients with non-otologic surgeries, surgeries performed outside primary institutions, and missing surgical time documentation were excluded. For clinical prediction modeling, a recommended rule of 10–20 observations per predictor variable suggests a target sample size of approximately 300 patients to ensure model stability and reduce overfitting. The final sample size of 216 patients, influenced by pandemic-related surgical volume reductions, is lower than the initially calculated requirement.

### 2.3 The variables and the data collection

Data were systematically extracted from electronic health records on demographic profiles, patient medical history, associated comorbidities, and both perioperative and intraoperative surgical variables. The surgical variables recorded were the surgical site, procedure type, and category of Surgery (classified as primary or revision, depending on surgical history and complexity). The intraoperative data includes the type of middle-ear approach, electrode type and insertion completeness, cochleostomy sealing technique, facial nerve monitoring, perioperative antibiotic regimen, and the use of preoperative systemic steroids. Facial nerve monitoring was used selectively based on surgeon preference and case risk profile and was not routinely applied in all procedures. Surgeons were categorized into consultants, fellows, and residents based on level of training and operative independence. Surgical time or operative time was defined as skin-to-skin time, meaning the interval from the initial skin incision to the placement of the final skin suture. It did not include anesthesia induction, patient preparation, positioning, draping, or any procedures performed before the skin incision or after wound closure. Total operating room time was defined as the interval from patient entry into the operating room until transfer out of the operating room.

### 2.4 Statistical analysis

Statistical analyses were performed using the Statistical Package for the Social Sciences (SPSS) version 26. (Armonk, New York, IBM Corporation, USA) and R software version 4.2.2. To address missing data, multiple imputations were conducted using the *mice* package in R. Imputation was performed under the assumption of missing at random (MAR), and five imputed datasets were generated and pooled for inference. Categorical variables were summarized as frequencies and percentages. Continuous variables were reported as mean ± SD or median [IQR], as appropriate. The Shapiro–Wilk test was used to assess the normality of continuous data distributions. Kruskal–Wallis tests were used to compare surgical time across demographic, clinical, and surgical variables with more than two groups, while Mann–Whitney U tests were applied to variables with two groups. Spearman's rank correlation was used to examine the association between surgical time and continuous variables. The study used a univariate generalized linear model to analyze the relationship between individual predictors and surgical time. A generalized linear model (GLM) was applied using a Gaussian distribution family with an identity link function for the continuous outcome (surgical time). Model assumptions were assessed to confirm suitability of this approach. Multicollinearity among predictors was evaluated using the variance inflation factor (VIF), with values >5 indicating significant collinearity. To minimize the risk of overfitting, only variables demonstrating clinical relevance and/or statistical association in univariate analyses were included in the multivariable GLM. A final predictive model was developed based on individual patient and surgical characteristics. The *p* value was considered significant if < 0.05.

## 3 Results

This study analyzed data from 216 CI recipients. Of these, 52.31% were male. The median age at hearing aid initiation was 12 months (interquartile range [IQR]: 6–24 months). Most patients (56.48%) had pre-lingual hearing loss. A positive family history of hearing loss was noted in 23.61% of cases, and 5.56% of patients were born prematurely. The median age at hearing loss onset was 0 months (IQR: 0–24), with a median duration of hearing aid use of 24 months (IQR: 8–84). The predominant indication for cochlear implantation was sensorineural hearing loss (SNHL), accounting for 91.67% of cases. The remaining cases (8.33%) included less common indications such as congenital syndromic hearing loss and acquired causes including post-infectious and traumatic hearing loss. Comorbidities were relatively uncommon and included meningitis in 0.46% (without specific imaging features) and inner ear anomalies (type II) in 5.56% of patients. Most patients received cefazolin as the preoperative antibiotic (95.83%), and steroids were administered preoperatively in 53.24% of cases.

Unilateral implantation was more frequently performed (59.72%) than bilateral CI. The vast majority of cases (93.52%) were primary surgeries. Facial nerve monitoring was utilized in 91.20% of the surgeries. The median total operating room time was 180 min (IQR: 150–240), while the median surgical time was 155 min (IQR: 120–185). Details of surgical techniques, electrode insertion approaches, intraoperative observations, and complications are provided in Table 1.

**Table 1:** Frequency distribution of demographic, clinical, and surgical characteristics of cochlear implant patients.

Characteristic	Value
Demographic variables	
Male sex, *n* (%)	113 (52.3)
Female sex, *n* (%)	103 (47.7)
Age at hearing aid initiation (months), median (IQR)	12 (6–24)
Language status, *n* (%)	
Pre-lingual	122 (56.5)
Peri-lingual	22 (10.2)
Post-lingual	72 (33.3)
Positive family history of hearing loss, *n* (%)	51 (23.6)
Consanguinity, *n* (%)	74 (34.3)
Prematurity, *n* (%)	12 (5.6)
Clinical variables	
Hearing loss onset (months), median (IQR)	0 (0–24)
Hearing aid use duration (months), median (IQR)	24 (8–84)
Sensorineural hearing loss, *n* (%)	198 (91.7)
Meningitis, *n* (%)	1 (0.5)
Inner ear anomaly, *n* (%)	12 (5.6)
Preoperative cefazolin use, *n* (%)	207 (95.8)
Preoperative steroid use, *n* (%)	115 (53.2)
Surgical variables	
Unilateral cochlear implantation, *n* (%)	129 (59.7)
Bilateral cochlear implantation, *n* (%)	87 (40.3)
Primary surgery, *n* (%)	202 (93.5)
Revision surgery, *n* (%)	11 (5.1)
Primary/revision surgery, *n* (%)	3 (1.4)
Facial nerve monitoring used, *n* (%)	197 (91.2)
Operating room time (minutes), median (IQR)	180 (150–240)
Surgical time (minutes), median (IQR)	155 (120–185)

IQR, interquartile range.

Surgical time was significantly longer among females (median: 180 min, IQR: 120–240) compared to males (median: 150 min, IQR: 120–180; *p* = 0.015). No significant associations were found with consanguinity, language background, prematurity, family history, or age at hearing aid use. Surgical time did not significantly correlate with age at hearing loss onset (*ρ* = 0.002, *p* = 0.978) or hearing aid duration (*ρ* = 0.078, *p* = 0.255). Additionally, clinical variables like indication (*p* = 0.567), meningitis (*p* = 0.916), or inner ear anomalies (*p* = 0.776) showed no significant effect on surgical time (Tables 2, 3).

**Table 2:** Comparison of surgical time among the demographic variables.

Variable	Surgical time(minutes)	*p*-value
Comparative groups	Median (Q1–Q3)	
Gender		
Male	150 (120–180)	**0.015**^b^*
Female	180 (120–240)	
Language		
Pre lingual	150 (120–180)	0.464^a^
Peri lingual	180 (135–210)	
Post lingual	170 (120–180)	
Positive family history		
No	160 (120–180)	
Yes	150 (120–190)	
Consanguinity		
No	173 (120–180)	0.824^a^
Yes	150 (120–190)	
Prematurity		
No	153 (120–180)	0.343^a^
Yes	180 (120–230)	

Relationship between surgical time and clinical variables		
Variables	Surgical time (minutes)	*p*-value
	Spearman s rank correlation (*ρ*)	
Age at hearing aids usage	0.054	0.430

a Kruskal Wallis test.

b Mann Whitneys u test.

*p value < 0.05 (significant).

**Table 3:** Comparison of surgical time among the clinical variables.

Variables	Surgical time (minutes)	*p*-value
	Median (Q1–Q3)	
Indication		
SNHL	150 (120–190)	0.567^a^
Hearing Loss (Not specified further)	180 (180–180)	
Post Meningitis SNHL	178 (175–180)	
Device Failure	85 (60–110)	
Bilateral Hearing Loss (not specified SNHL)	180 (120–180)	
Bilateral Cochlear Implant	148 (100–195)	
Other (Miscellaneous)	145 (110–180)	
Kernicterus		
No	150 (120–190)	0.677^b^
Yes	160 (120–180)	
Meningitis		
No	155 (120–190)	0.916^b^
Yes	175 (175–175)	
Temporal bone fracture		
No	155 (120–185)	NA
Yes	0 (0–0)	
Inner ear anomaly		
No	153 (120–180)	0.776^b^
Yes	160 (69–225)	
Cochlear ossification		
No	153 (120–180)	0.478^b^
Yes	183 (175, 190)	
Pre-op antibiotic Type		
Cefazolin	155 (120–180)	0.319^b^
Cefuroxime	210 (150–240)	
Pre-op Steroid		
No	150 (110–190)	0.078^b^
Yes	180 (120–180)	

Relationship between surgical time and clinical variables		
Variables	Surgical time (minutes)	*p*-value
	Spearman s rank correlation (*ρ*)	
Hearing loss onset (months)	0.002	0.978
Hearing aid use duration (months)	0.078	0.255

SNHL, sensorineural hearing loss.

a Kruskal Wallis test.

b Mann Whitneys u test.

The study analysed surgical time across various variables, revealing significant differences between different types of surgeries, surgeons, skin incision types, insertion techniques, quality of insertion, sealing of the insertion route, and material used (*p* < 0.05). Type of surgery significantly impacted the surgical time (190 min vs. 120 min; *p* < 0.001), while Revision surgeries had shorter median surgical times compared to combined primary/revision cases (110 min vs. 240 min, *p* = 0.003). Surgical time differed significantly across surgeon groups, with residents showing the longest duration, followed by fellows and consultants **(*p*** **=** **0.000).** Use of the round window (RW) approach had shorter times than cochleostomy **(*p*** **=** **0.036).** Clear insertions had longer durations than complete insertions **(*p*** **=** **0.017)** and sealing the insertion route was associated with shorter times **(*p*** **=** **0.025).** Among continuous variables, operating room time in minutes showed a significant positive correlation with surgical duration **(*ρ*** **=** **0.660, *p*** **=** **0.000)** (Table 4).

**Table 4:** Comparison of surgical time among the surgical variables.

Variables	Surgical time (minutes)	*p*-value
	Median (Q1–Q3)	
Type of surgery		
Unilateral CI	120 (110–180)	**0.000**^b^*
Bilateral CI	190 (150–250)	
Site		
Primary	160 (120–190)	**0.003**^a^*
Revision	110 (90–135)	
Primary/Revision	240 (195, 300)	
Facial Monitor Usage		
No	155 (150–240)	0.369^b^
Yes	155 (120–180)	
Surgeon		
Fellow	150 (110–190)	**0.000**^a^*
Fellow/resident	193 (150–260)	
Consultant	120 (100–170)	
Consultant/ fellow	180 (180–180)	
Consultant/resident	135 (120–150)	
Consultant/fellow/ Resident	180 (120–180)	
Skin incision type		
Lazy-s	133 (100–180)	**0.024**^b^*
Sulculus	163 (120–195)	
Skinincision_size		
C-shaped	240 (240–240)	0.640^b^
Small (1–3 cm)	150 (120–185)	
Postauricular – (Small1 cm post auricular incision away from)	170 (160–180)	
Superiorly based flap	175 (175–175)	
Periosteal flap		
Palva	160 (120–200)	0.299^a^
Superiorly based	150 (120–180)	
T- shaped	180 (135–180)	
Mixed(Superiorly based + T-shaped)	120 (120–120)	
Implanted well		
Dural exposed	178 (120–180)	0.094^a^
Distance from the post wall of EAC	135 (110–180)	
Not needed	148 (90–195)	
Approach to ME		
Facial recess	150 (120–180)	0.198^b^
Other Approaches	180 (180–240)	
Facial N. & chorda tympani		
Identified	155 (120–180)	0.426^b^
Other/Not Fully Identified	180 (150–210)	
Insertion		
RW	150 (120–180)	**0.036**^b^*
Cochleostomy	180 (145–218)	
Quality of the insertion		
Clear	175 (120–180)	**0.017**^a^*
Complete	120 (100–180)	
Partial	225 (150–280)	
Difficult	120 (120–135)	
Mixed	240 (240–240)	
Others	240 (240–240)	
Round Window		
Clear	180 (120–195)	0.495^a^
Clear/ required niche removal	183 (120–285)	
Required niche removal	150 (120–180)	
Sealing the insertion route		
No	180 (120–200)	**0.025**^b^*
Yes	140 (120–180)	
Material used		
Fascia	143 (120–180)	**0.038**^a^*
Muscle	120 (70–145)	
Fascia + muscle	180 (180–180)	
Synthetic Material (flex 26, Stupper)	150 (150–180)	
Not specified	180 (120–200)	
Intraoperative complications		
Dural exposure	210 (180–210)	0.328^a^
dural exposure/injury to sigmoid sinus	140 (100–180)	
Dural exposure (reappeared with cartilage)	150 (150–150)	
CSK leak/Dural Exposure	180 (180–180)	
Injury to sigmoid sinus	60 (60–60)	
Granulation tissue over the area of round window	120 (120–120)	
None/No complications	155 (120–180)	
Dural Exposure		
No	150 (120–180)	0.223^b^
Yes	180 (150–210)	
Sigmoid Sinus Injury		
No	155 (120–190)	0.443^b^
Yes	120 (60–180)	
Gusher		
No	155 (120–190)	NA
Yes	0 (0–0)	
Granulation Tissue	–	
No	155 (120–190)	0.452^b^
Yes	120 (120–120)	
None/no complications		
No	180 (120–210)	0.645^b^
Yes	155 (120–180)	
Subcutaneous		
Vicryl 3-0	150 (120–180)	0.793^a^
Vicryl 4-0	155 (110–205)	
Vicryl 2-0	180 (135–240)	
Other Vicryl	180 (120–180)	
Three-layer closure	120 (120–120)	
Two-layer closure	135 (135–135)	
Interrupted/Other methods	105 (105–105)	

Variables	Surgical time (minutes)	*P*-value
	Spearman s rank correlation (*ρ*)	
OR time (Months)	0.660	**0** **.** 000**
Wall of EAC mm	0.040	0.563
With angle (°)	0.029	0.670

Post hoc comparisons revealed significant differences between site groups (Primary vs. Revision: *p* = 0.007; Primary vs. Primary/Revision: *p* = 0.033; Revision vs. Primary/Revision: *p* = 0.010); among surgeon categories (Fellow vs. Fellow/Resident: *p* = 0.002; Fellow/Resident vs. Consultant: *p* = 0.000; Fellow/Resident vs. Consultant/Resident: *p* = 0.000; Consultant vs. Consultant/Fellow: *p* = 0.007; Consultant vs. Consultant/Fellow/Resident: *p* = 0.019; Consultant/Fellow vs. Consultant/Resident: *p* = 0.001; Consultant/Resident vs. Consultant/Fellow/Resident: *p* = 0.015); for quality of insertion (Clear vs. Complete: *p* = 0.021); and for material used (Muscle vs. Not Specified: *p* = 0.005).

OR, Operative time; EAC, external auditory canal.

a Kruskal Wallis test.

b Mann Whitneys u test.

*p value < 0.05 (significant).

**p < 0.001 (significant).

After applying the Bonferroni correction for multiple comparisons, only three variables remained statistically significant. Type of surgery (adjusted *p* < 0.001), Surgeon (adjusted *p* < 0.001), and OR time (adjusted *p* < 0.001) retained significance in the univariate analysis, indicating robust associations with surgical time. These corrections were applied solely to the exploratory univariate comparisons to control the error rate. In contrast, other variables that were initially significant before adjustment, including Gender (unadjusted *p* = 0.015; adjusted *p* = 0.555), HL onset (unadjusted *p* = 0.002; adjusted *p* = 0.074), Site (unadjusted *p* = 0.003; adjusted *p* = 0.111), Skin incision type (unadjusted *p* = 0.024; adjusted *p* = 0.888), Insertion (unadjusted *p* = 0.036; adjusted *p* = 1.000), Quality of insertion (unadjusted *p* = 0.017; adjusted *p* = 0.629), Sealing the insertion route (unadjusted *p* = 0.025; adjusted *p* = 0.925), and Material used (unadjusted *p* = 0.038; adjusted *p* = 1.000), did not remain significant after adjustment. The remaining values also remained insignificant, and applying the Holm correction yielded the same set of significant variables. This consistency underscores the robustness of the identified associations while highlighting the importance of adjusting for multiple comparisons to reduce the risk of false-positive findings.

Variables included in the multivariable generalized linear model were selected based on clinical relevance and statistical evidence from the univariate analyses. Multivariate regression revealed that male gender was associated with reduced surgical time by 16.6 min, CI 95% (−0.36, −32.77) (*p* = 0.0464); unilateral CI shortened duration by nearly 62 min (*p* < 0.00001); and round window insertion reduced time by 28.5 min (*p* = 0.0445). Older surgical age was associated with longer duration (0.13 min/year; *p* = 0.0034) (Table 5).

**Table 5:** Predictors of surgical time: a multivariate regression analysis.

Predictor	Estimate	Std. Error	T value	*P* value	Interpretation
(Intercept)	205.28	21.30	9.64	**<0** **.** 00001**	Baseline surgical time when all variables are at reference level.
Gender (male)	−16.57	8.27	−2.00	**0** **.** 0464*	Male patients had significantly shorter surgical times by ∼16.6 min.
Skin incision (sulculus)	12.69	10.43	1.22	0.225	Not significant; suggests slightly longer times with sulculus incision.
Type of surgery (Unilateral CI)	−61.80	10.18	−6.07	**<0** **.** 00001**	Strongly significant; unilateral CI reduces surgery time by ∼62 min.
Surgeon Consultant/Fellow	28.68	17.43	1.65	0.101	Not statistically significant; possible trend toward longer times.
Surgeon Consultant/Fellow/Resident	43.33	16.43	2.64	**0** **.** 0090**	Significant; this combination increases surgery time by ∼43 min.
Surgeon Consultant/Resident	6.54	17.21	0.38	0.704	Not significant.
Surgeon Fellow	6.00	14.17	0.42	0.672	Not significant.
Surgeon Fellow/Resident	26.44	16.87	1.57	0.119	Not significant; trend toward increased time.
Insertion method (RW)	−28.48	14.08	−2.02	**0** **.** 0445*	Statistically significant; RW reduces surgical time by ∼28.5 min.
Age at surgery (Hausage)	0.13	0.044	2.96	**0** **.** 0034**	Statistically significant; older age is associated with slightly longer surgery time.

*p < 0.05; **p < 0.001.

The regression equation to estimate surgical time is:

$$\begin{array}{rl} & Surgical\;Time\;(min)=\beta_{0}+\beta_{1}\times Gender+\beta_{2}\times CI\;Type\\ & +\beta_{3}\times Surgeon\;Level+\beta_{4}\times SurgicalApproach+\beta_{5}\times Age \end{array}$$

$$\begin{array}{rl} & SurgicalTime=205.28-16.57\times Male-61.80\\ & \times UnilateralCI+43.33\times Consultant/Fellow/Resident\\ & -28.48\times RWInsertion+0.13\times Ageatsurgery \end{array}$$

Each variable here represents a predictor of surgical time (in minutes), and the coefficient shows how much that variable changes the predicted surgical time, as shown in Supplementary Table.

After generalization, the predictive formula for cochlear implant surgical time becomes more interpretable and applicable across different settings. In this model, males are expected to require approximately 15 min less surgical time compared to females. Unilateral cochlear implantation is significantly quicker, reducing surgical time by about 60 min compared to bilateral procedures. The surgeon's level of experience also plays a key role; each step down in experience (from consultant to fellow to resident) adds roughly 20 min to the surgery duration. Additionally, using the RW insertion technique instead of cochleostomy shortens the operation by approximately 25 min. Lastly, the patient's age has a small effect, with each additional year increasing the surgical time by around 0.15 min (Table 6).

$$\begin{array}{rl} & Generalized\;Formula\;(Approximate\;Coefficients)\;Surgical\;Time\\ & =200-15\times (Male)-60\times (UnilateralCI)+20\\ & \times (SurgeonLevel)-25\times (RWInsertion)+0.15\times (Age) \end{array}$$

**Table 6:** Generalized predictive equation components and their effects on operative time.

Variable	Coefficient	Interpretation
Male	−15	Males take ∼15 min less than females
Unilateral CI	−60	Unilateral CI is ∼60 min faster than bilateral CI
Surgeon Level	+20	Each level lower in experience adds ∼20 min
RW Insertion	−25	Round window approach reduces time by ∼25 min
Age	+0.15	Each year of age adds ∼0.15 min to surgery time

## 4 Discussion

This study uncovers a set of variables that significantly affect skin-to-skin time by analyzing 216 consecutive cases through applying rigorous univariable non-parametric tests, followed by a univariate GLM to assess the association between individual predictors and surgical time. Using non-parametric tests and a generalized linear modeling framework, we evaluated both individual and adjusted effects of key predictors on operative duration.

Key predictors of surgical time include gender, type of surgery, surgeon experience, method of insertion, and age at the time of surgery. Previous studies from Saudi Arabia reported several factors affecting CI operative time, with complications such as otitis media prolonging surgery by ∼45 min, though independent effects were unclear due to unstratified analyses (9). Hajr et al. found that the CR220 system shortened intraoperative testing but did not assess total surgical time, limiting generalizability (10). Similarly, a pediatric study showed ECAP and impedance testing added ∼6.7 min, unaffected by audiologist expertise or electrode type (11). Our study expands on these by modeling total operative time, including intraoperative steps, surgeon variability, and patient factors.

Type of surgery was one of the strongest predictors of operative time and remained significant in multivariable analysis, reflecting the increased complexity associated with more extensive surgical exposure. Previous studies have confirmed that bilateral CI takes significantly longer than unilateral procedures (12). The majority of studies found that simultaneous bilateral cochlear implantation has a shorter surgical time than consecutive cochlear implantation (13). A retrospective study found bilateral CI reduced cumulative surgical and inpatient time compared to sequential unilateral implantation (14). Our model estimates unilateral implantation reduces operative duration by ∼40% (−61.8 min), emphasizing the significant planning impact of laterality.

Procedures involving trainees added ∼43 min compared to consultant-only teams, consistent with reports of longer operative times in teaching cases despite unchanged outcomes (15). Training remains essential, but scheduling should account for trainee-related delays, and simulation-based strategies may help recover time without compromising educational value (16).

The round window (RW) approach reduced operative time by ∼28.5 min (*p* = 0.0445), aligning with studies suggesting RW insertion is generally less invasive and faster than cochleostomy, particularly in well-visualized cases (17). In our center, cochleostomy or an extended RW is used when visualization is limited (18). Anatomical variations, such as post-meningitic cochlear ossification, can obscure RW and occasionally lead to incomplete insertion, as reported in five of fifteen infant cases in a retrospective series by Hajr et al. (19). Thus, while RW offers efficiency, patient-specific factors must guide access planning. Sulcus skin incisions were also linked to longer procedures (*p* = 0.0225), whereas sutureless tunnel methods have shown time savings (20). Complex anatomy, revision surgery, and infant mastoids with high marrow content further necessitate meticulous dissection, potentially prolonging surgery and increasing the risk of complications like facial nerve injury (21).

Interestingly, the multivariable model showed that patients with each additional year of age had an increase in the operative duration by 0.13 min (*p* = 0.0034). Older age-related surgical risk needs to be assessed before surgical procedures, as they continue to experience adverse effects like morbidity and mortality risk, extended operative time, and length of stay (22).

The multivariate analysis revealed that surgeries in male patients were, on average, 16.6 min with CI 95% (−0.36, −32.77) shorter than those in female patients (*p* = 0.0464). In contrast to the other studies that reported no significant association between gender and operative time (23). While modest sex-related differences in cochlear duct length and temporal bone morphology have been reported (24), there is no evidence that these variations affect cochlear implant operative time, and no studies have specifically evaluated sex as a predictor of operative duration. Therefore, the observed association may reflect anatomical or workflow-related factors, such as trainee involvement, but its underlying mechanism remains unclear. Further research is needed to clarify this relationship.

Several factors lost significance after multivariate adjustment, including PPI prophylaxis, facial nerve monitoring, preoperative steroids, age of hearing loss onset, and antibiotic choice. Cefazolin was the standard prophylaxis, with cefuroxime reserved for complex cases, likely explaining minor observed variations (25). Facial nerve monitoring, performed concurrently with the procedure, did not meaningfully prolong operative time, and historical concerns that cochlear ossification or inner-ear anomalies necessitate prolonged drilling were not supported, likely due to improved imaging and surgical expertise (26). Identifying non-contributory variables allows quality- improvement efforts to focus on factors with the greatest impact on efficiency.

Only five factors, adult age, surgery, unilateral status, male gender, surgeon combination, skin incision, and RW insertion, accounted for a significant portion of the variation in duration. The current equation is directly calculable and can therefore be integrated into electronic scheduling dashboards, in contrast to Bennett's previous model, which used ANOVA categories instead of regression (27). The model's simplicity, clinical interpretability, and direct applicability to surgical scheduling represent important strengths over complex machine-learning algorithms, despite their potential for accuracy. It's also more consistent than many retrospective studies. Using surgeon CPT and demographic data, machine learning projects in orthopedics and general surgery report mean absolute errors of 10%–20% in case length prediction (28). Despite its simplicity, the current model may be as good as or better than black-box approaches for CI scheduling because it focuses on domain-specific drivers that aren't present in generic hospital datasets. To support clinical application of this model, we developed a bilingual (Arabic/English) web-based calculator using the final predictive equation. This tool enables clinicians to rapidly estimate cochlear implant surgical time based on patient and procedural variables **(online calculator; see** Supplementary Material **for access details)**.

### 4.1 Strengths and limitations

This single-center retrospective study has several strengths, including detailed perioperative data capturing anatomical, procedural, and staffing variables. However, this study has several limitations. First, it was conducted at a single tertiary academic institution, which may limit the generalizability of the findings to other healthcare settings with different referral patterns and surgical practices. Second, the retrospective design relies on the accuracy and completeness of electronic health records and is subject to potential bias. Although surgeon experience level was included, other surgeon-related variables such as the number of surgeons involved, surgical volume, and years of experience were not captured, despite their potential influence on operative duration. The final sample size was smaller than initially calculated due to pandemic-related surgical reductions, which may have limited the statistical power to detect smaller effects. Although the predictor-to-sample ratio was acceptable, the model was developed and tested within the same dataset. Internal validation techniques such as bootstrapping or cross-validation were not performed, and therefore some degree of model optimism cannot be excluded. Furthermore, while multiple demographic, clinical, and intraoperative factors were analyzed, unmeasured confounders such as intraoperative complications, assistant surgeon involvement, or workflow variations may have affected operative time. Finally, although multiple imputation was applied to address missing data under the assumption of missing at random, any deviation from this assumption could introduce bias into the findings.

Future studies should validate findings in other centers, refine surgeon-related coefficients, and integrate anesthesia workflow and OR logistics. Testing interventions like dedicated bilateral pathways and exploring female-associated prolongation, patient-reported experiences, and early audiologic outcomes will clarify the impact of efficiency on care quality.

## 5 Conclusion

This study provides an internally derived predictive model and a thorough analysis of the factors influencing surgical duration in cochlear implantation. The findings show that operating times are largely affected by gender, age, unilateral implantation, the surgeon combinations, skin incision, and RW technique. The predictive model should be considered internally derived and requires external validation before routine clinical implementation.

## Data Availability

The original contributions presented in the study are included in the article/Supplementary Material, further inquiries can be directed to the corresponding author.

## Supplementary material

Supplementary Table S1 Significant variables coding and description.

Supplementary Table S2 Predictive equation components and their effects on operative time.
