# Supplementary material for: A predictive equation for operative time estimation in cochlear implant surgery
Source: Front Surg. 2026 Jul 13;13:1857136. doi: 10.3389/fsurg.2026.1857136 (PMC13402822; doi:10.3389/fsurg.2026.1857136)
Supplement: Supplementary Table S1 — Significant variables coding and description. [file Supplementaryfile1.docx]

**Table SS: Significant Variables Coding and Description**

| **Variable** | **Description** | **Coding** |
| --- | --- | --- |
| **Gender** | Male = 1, Female = 0 | Binary |
| **CI Type** | Unilateral = 1, Bilateral = 0 | Binary |
| **Surgeon Level** | Consultant = 0, Fellow = 1, Resident = 2 | Ordinal or dummy |
| **Surgical Approach** | RW = 0, Cochleostomy = 1 | Binary |
| **Age** | Age in years | Continuous |

**Table 1S: Predictive Equation Components and Their Effects on Operative Time**

| Variable | Coefficient | Effect on Operative Time |
| --- | --- | --- |
| Male gender | -16.57 | Decreases by 16.6 minutes |
| Unilateral CI | -61.80 | Decreases by 61.8 minutes |
| Consultant/Fellow/Resident involvement | +43.33 | Increases by 43.3 minutes |
| Round Window insertion | -28.48 | Decreases by 28.5 minutes |
| Age (per year) | +0.13 | Increases by 0.13 minutes per year |
